# Supplementary material for: Influence of the COVID-19 Pandemic on Adherence to Orally Administered Antineoplastics
Source: J Clin Med. 2022 Apr 26;11(9):2436. doi: 10.3390/jcm11092436 (PMC9103306; doi:10.3390/jcm11092436)
Supplement: Supplementary file 1 [file jcm-11-02436-s001.zip › jcm-1649330-supplementary.pdf]

Table S1. Description of the orally administered antineoplastics included in the study.

ORALLY ADMINISTERED ANTINEOPLASTICS	N	%
**IMMUNOMODULATORS**	**44**	**16.4**
LENALIDOMIDE	41	15.3
POMALIDOMIDE	*3*	*1.1*
**CYTOTOXIC CHEMOTHERAPY**	**34**	**12.7**
CAPECITABINE	*25*	*9.3*
TEMOZOLOMIDE	*3*	*1.1*
TRIFLURIDINE + TIPIRACIL	*4*	*1.5*
LOMUSTINE	*1*	*0.4*
VINORELBINE	*1*	*0.4*
**DIRECTED THERAPIES**	**186**	**72.4**
IBRUTINIB	*29*	*10.8*
ABIRATERONE	*19*	*7.1*
RIBOCICLIB	*16*	*6*
PALBOCICLIB	*10*	*3.7*
ENZALUTAMIDE	*9*	*3.4*
RUXOLITINIB	*9*	*3.4*
IMATINIB	*8*	*3*
NILOTINIB	*8*	*3*
OLAPARIB	*8*	*3*
OSIMERTINIB	*8*	*3*
DASATINIB	*6*	*2.2*
NINTEDANIB	*6*	*2.2*
SUNITINIB	*6*	*2.2*
ABEMACICLIB	*5*	*1.9*
ALECTINIB	*4*	*1.5*
DABRAFENIB + TRAMETINIB	*4*	*1.5*
CABOZANTINIB	*3*	*1.1*
EVEROLIMUS	*3*	*1.1*
NIRAPARIB	*3*	*1.1*
PAZOPANIB	*3*	*1.1*
SORAFENIB	*3*	*1.1*
LAPATINIB	*2*	*0.8*
REGORAFENIB	*2*	*0.8*
VENETOCLAX	*2*	*0.8*
VISMODEGIB	*2*	*0.8*
AFATINIB	*1*	*0.4*
ALPESINIB	*1*	*0.4*
AXITINIB	*1*	*0.4*
ENCORAFENIB	*1*	*0.4*
LENVATINIB	*1*	*0.4*
PONATINIB	*1*	*0.4*

TIVOZANIB	*1*	*0.4*
VANDETANIB	*1*	*0.4*
**OTHER**	**4**	**1.5**
ANAGRELIDE	*4*	*1.5*
**TOTAL**	**268**	**100**
